# A Voxel-Wise Meta-Analysis of Gray Matter Abnormalities in Essential Tremor

**DOI:** 10.3389/fneur.2018.00495

**Published:** 2018-06-26

**Authors:** Qing Han, Yanbing Hou, Huifang Shang

**Affiliations:** Department of Neurology, West China Hospital, Sichuan University, Chengdu, China

**Keywords:** essential tremor, voxel-based morphometry, meta-analysis, gray matter, structural abnormality, default mode network

## Abstract

**Objective:** To identify the consistent gray matter (GM) volume changes from the whole brain voxel-based morphometry (VBM) studies on essential tremor (ET).

**Methods:** The whole brain VBM studies comparing ET patients and healthy controls (HCs) were systematically searched in the PubMed, Embase and Web of Science from January 2000 to December 2017. Coordinates with significant differences in regional GM volume between ET patients and HCs were extracted from included studies and the meta-analysis was performed using effect size-based signed differential mapping (ES-SDM).

**Results:** A total of 10 studies with 241 ET patients and 213 HCs were included in the meta-analysis. The consistent GM volume reduction was detected in the left precuneus extending to the left posterior cingulate gyrus. The subgroup meta-analysis which included studies performed on a 3.0 T scanner revealed significant GM volume increases in the bilateral frontal lobes, bilateral temporal lobes, left insula, left striatum and left pons, but obvious publication biases of these findings were detected through funnel plots and Egger's tests.

**Conclusions:** The consistent result of our meta-analysis showed a structural damage in the left precuneus extending to the left posterior cingulate gyrus, which possibly played a role in the cognitive dysfunction and depression in ET patients. It might enhance our understanding of the pathophysiological mechanisms underlying ET.

## Introduction

Essential tremor (ET) is one of the most common movement disorders, usually characterized by largely symmetrical postural and kinetic tremor of the upper limbs in the absence of other neurological signs (1). Recently, it is generally considered that ET encompasses a range of heterogeneous clinical phenotypes, as some patients manifest more extensive and complex deficits (2). A lot of efforts have been spent in the pathological, neuroimaging, physiological and clinical researches, which pointed that ET may be a neurodegenerative disorder mainly involving cerebellum (3–6). However, the specific pathophysiological mechanism of ET remains poorly understood and controversial (7).

Among different kinds of studies aimed to reveal the pathophysiology of ET, the structural neuroimaging study is important and basic for the *in vivo* exploration of brain abnormalities. Voxel-based morphometry (VBM), the most common method in structural neuroimaging studies, is a processing technique of magnetic resonance imaging (MRI) that can detect regional morphological changes in the whole brain. During the last 20 years, a number of VBM studies detected the gray matter (GM) differences between ET patients and healthy controls (HCs) but the findings of these studies are variable and conflicting. The most prevalent findings were GM abnormalities in the cerebellum. Cerebellar GM volumes reductions of ET patients were detected widely in vermis, anterior and posterior lobules (8–12) while increased regional cerebellar GM volumes were also reported (10). In addition, the GM volumes abnormalities were found in various cerebral regions located in temporal lobe, frontal lobe, parietal lobe, precuneus, insula, caudate nucleus and so on, which mainly indicated motor networks potentially affected in ET like the cerebello-thalamo-cortical network (8–10, 12, 13). However, there were quite a few studies reported no significant difference of GM volume changes between general ET patients and HCs (14–18).

A previous review (4) aimed to structural and functional neuroimaging studies of ET has well summarized the findings of many VBM studies but did not performed a qualitative or quantitative analysis. Therefore, it is timely to conduct a meta-analysis to identify the most consistent regional GM volume changes in ET. The effect size-based signed differential mapping (ES-SDM), a quantitative voxel-based meta-analytic tool for neuroimaging studies that has been widely used in various neurodegenerative and neuropsychiatric diseases (19–22), was apply to the present study.

## Methods

### Data sources, study inclusion, and data extraction

A comprehensive search of studies published from January 2000 to December 2017 was conducted in the PubMed, Embase and Web of Science using the combined keywords (“essential tremor” OR “ET”) and (“voxel-based” OR “VBM” OR “morphometry”). An additional search was also conducted in the reference list of relevant articles.

Studies were considered for inclusion if they (a) investigated GM volume differences between ET patients and HCs using whole-brain voxel-based analysis; (b) did not find significant differences or reported significant results in a standard stereotactic space (Talairach or Montreal Neurological Institute [MNI]) with three-dimensional coordinates (x, y, z); (c) were peer-reviewed and published in English. Studies were excluded if (a) stereotactic coordinates of the reported changes in the whole brain were not obtained even if we contacted with corresponding authors by email; (b) studies limited their analysis to specific regions of interest (ROI); (c) studies only reported significant results of subgroup analyses comparing specific ET patients with HCs; (d) studies used different thresholds in different regions of the brain. The latter criterion is intended to avoid biases toward liberally thresholded brain regions (19). For those studies that met the aforementioned inclusion criteria with overlapping samples, only the study with the largest sample size was included to avoid repetitive data.

The quality of each study selected for this meta-analysis was assessed with a 15-point checklist (Supplementary Table 2) which focused on demographic aspects of samples, imaging methodology and statistical analysis. This checklist was based on previous imaging meta-analyses which considered structural and functional measures from MRI (23, 24), but modified to reflect important variables for the current meta-analysis.

In each included study, we chose the results from general ET patients comparing with HCs instead of those from selected patients within subgroup analyses to avoid selection bias. The coordinates and their effect sizes (*t* statistics, *z* scores or *p* values) with significant differences between ET patients and HCs were extracted according to the AES-SDM software tutorial.

The literature search, study selection and data extraction were all performed by two neurologists independently. If there were disagreements, the third neurologist would check the data and make a decision finally. The Meta-analysis Of Observational Studies in Epidemiology (MOOSE) guidelines for the meta-analyses of observational studies were followed in the current study.

### Meta-analysis

The meta-analysis of included studies was performed in a standard process using the SDM software package (www.sdmproject.com) to compare the differences of regional GM volumes between ET patients and HCs. The detailed SDM approach has been described elsewhere previously (25–27). In brief, a file containing the coordinates, effect size, sample sizes and other clinical data (e.g., mean age and disease duration of ET patients) was established for each study firstly. If an included study did not find significant coordinates, we also created a file containing the above data and marked it “no peaks.” The SDM software would extract data from these files and recreate an effect-size map of the regional GM volume differences for each study. The mean map was then produced through a meta-analytical random-effects model where the weight of each study was calculated by the sample size, intra-study variability and between-study heterogeneity. Finally, the statistical significance was established using standard randomization tests. The default kernel size and statistical thresholds (full width at half maximum [FWHM] = 20 mm, *p* = 0.005, peak height threshold = 1, extent threshold = 10) were used to optimize the recreation of the effect size maps but also keep it robust (28). A subgroup meta-analysis would be conducted additionally if necessary.

Jackknife sensitivity analyses were performed to assess robustness of findings by repeating the mean analysis excluding one study each time to see whether findings remained significant. In addition, a heterogeneity analysis was carried out to determine significant unexplained between-study variability within the results using a random effects model with Q statistics (21, 27). Moreover, to examine potential publication bias, we extracted values from statistically significant relevant peaks and then conducted funnel plots for visual inspection (29) and Egger's tests (*P* < 0.05 was considered significant) for more accurate assessment (30) using the SDM software package.

Finally, we carried out meta-regression analyses to examine effects of age, duration of disease, illness severity and head tremor, which might influence the results of the meta-analysis. The mean age, mean illness duration, mean Fahn-Tolosa-Marin tremor rating scale (TRS) scores and percentage of ET patients with head tremor were extracted from each included study and the default statistical threshold (*p* = 0.0005 and cluster extent = 10 voxels) (28) was used as well.

## Results

### Included studies and sample characteristics

After initially searching the titles and abstracts with the search strategy, 13 VBM studies (8–18, 31, 32) were identified as potentially suitable. A detailed review of the full text was then performed and 3 studies were excluded: one study (13) did not report stereotactic coordinates; one study (31) used voxel-based MRI T2^*^-relaxometry to detect iron concentration in the whole brain instead of regional GM volume changes; one study (32) only reported significant results of subgroup analyses of ET patients with impaired cognition. Finally, 10 studies (8–12, 14–18) were included in the meta-analysis, comprising 241 ET patients and 213 HCs, which were matched to age in all included studies. Of the 10 studies, seven (9, 11, 12, 14–17) enrolled patients diagnosed as at least probable ET according to the Movement Disorders Consensus Criteria (1); one (18) enrolled patients based on a more detailed criterion defined by the Tremor Investigation Group (33); one (8) enrolled patients based on another published research criteria (34); and one (10) did not report the diagnosis criteria. In addition, eight out of the 10 studies were performed on a 3.0 T scanner and two studies (14, 15) were performed on a 1.5 T scanner. Clinical and imaging characteristics of the 10 studies are summarized in Table 1.

**Table 1:** Characteristics of included VBM studies in the current meta-analysis.

**Study**	**Sample**	**Sex (M/F)**	**Mean age (years)**	**Duration (years)**	**TRS scores**	**Software**	**Threshold**	**Scanner**	**h-ET**	**Quality scores**
(14)	ET 27	18/9	57.9 ± 12.2	26.0 ± 17.5	33.9 ± 13.7	SPM2	*P* < 0.05 corrected	1.5T	NA	15
	HC 27	18/9	57.6 ± 11.6							
(15)	ET 50	24/26	65.2 ± 14.3	19.3 ± 14.9	NA	SPM2	*P* < 0.001 uncorrected	1.5T	20	13
	HC 32	16/16	66.2 ± 8.1							
(8)	ET 19	10/9	69.8 ± 9.4	11.0 ± 6.8	NA	SPM5	*P* < 0.001 uncorrected	3.0T	11	13
	HC 20	10/10	68.9 ± 10.0							
(9)	ET 20	15/5	38.2 ± 16.5	4.7 ± 4.5	29.7 ± 14.9	SPM5	*P* < 0.001 uncorrected	3.0T	10	14
	HC 17	14/3	40.7 ± 16.5							
(10)	ET 10	5/5	63.4 ± 8.7	15.2 ± 7.91	NA	SPM8	*P* < 0.001 uncorrected	3.0T	NA	13
	HC 13	9/4	65.3 ± 11.1							
(16)	ET 14	9/5	61.2 ± 12.0	13.2 ± 11.9	17 ± 7.3	SPM5	NA	3.0T	0	13
	HC 20	NA	60.2 ± 8.1							
(11)	ET 14	8/6	64.4 ± 9.1	12.8 ± 11.9	10.1 ± 4.6	SPM8	*P* < 0.001 uncorrected	3.0T	NA	14
	HC 23	13/10	64.4 ± 7.1							
(12)	ET 19	12/7	50.4 ± 15	21.3 ± 13.7	38.9 ± 20.8	SPM8	*P* < 0.05 corrected	3.0T	NA	15
	HC 19	12/7	50.1 ± 16.4							
(17)	ET 32	17/15	69.7 ± 9.7	13.4 ± 12.9	25.6 ± 9.5	FSL	*P* < 0.05 corrected	3.0T	NA	15
	HC 12	4/8	67.4 ± 4.8							
(18)	ET 36	23/13	56 ± 14	27 ± 16	NA	SPM8	NA	3.0T	13	13
	HC 30	19/11	54 ± 15							

*VBM, voxel-based morphometry; ET, essential tremor; HC, healthy control; M/F, Male/Female; TRS, Fahn-Tolosa-Marin tremor rating scale; h-ET, ET patients with head tremor; NA, none reported. Mean±standard deviation*.

### Regional differences in GM volume

**Figure 1:**
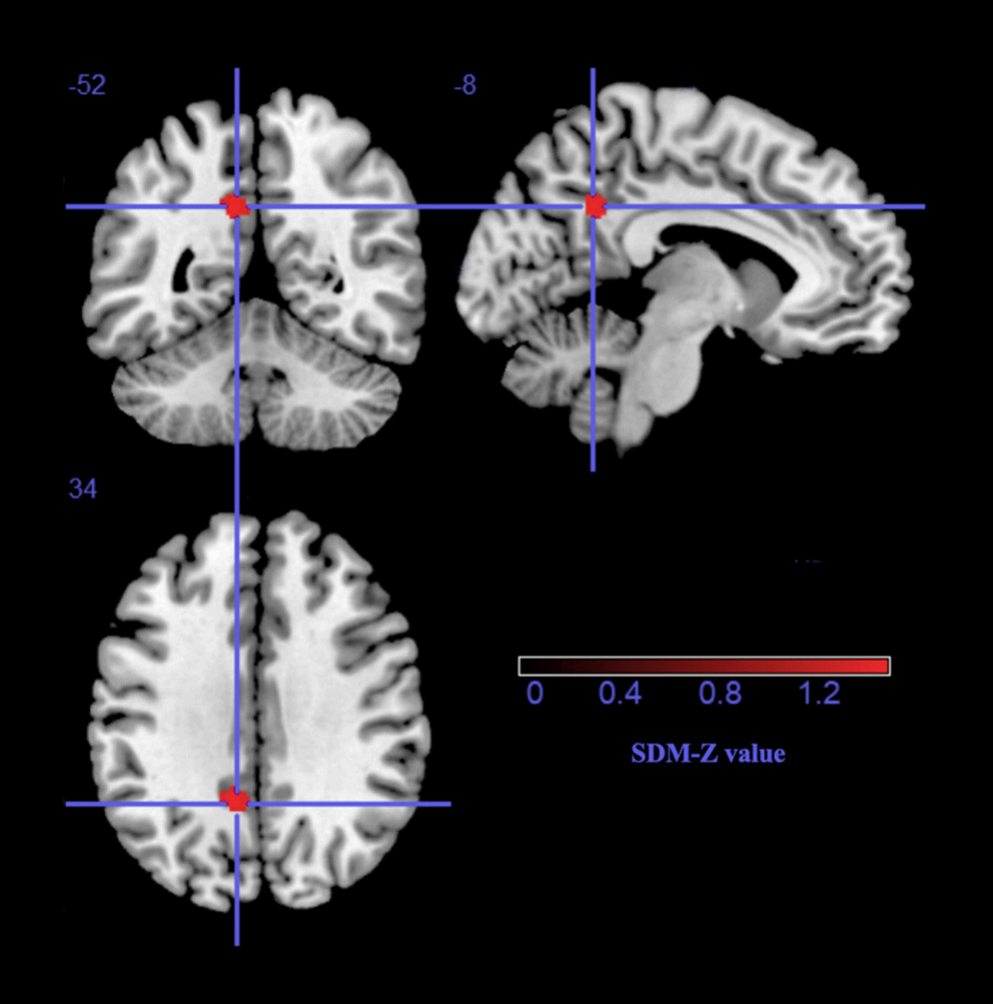
The gray matter volume reduction in the left precuneus extending to the left posterior cingulate gyrus, BA 23. SDM, Seed-based d Mapping.

As shown in Table 2 and Figure 1, a significant regional GM volume decrease was detected in the left precuneus (BA23) extending to the left posterior cingulate gyrus (BA23) in ET patients from the whole meta-analysis. No significant regional GM volume increase in ET patients was found in any region compared with HCs.

**Table 2:** The main meta-analysis: Gray matter volume reductions in ET patients relative to healthy controls.

**Regions**	**No. of voxels**	**Maximum MNI coordinates (x,y,z)**	**SDM-Z value**	***P*-value**	**Jackknife sensitivity**	**Egger test (*p*-value)**
L precuneus/L posterior cingulate gyrus, BA23	69	−8,−52, 34	−1.471	0.002	8 out of 10	0.043

*ET, essential tremor; No., number; MNI, Montreal Neurological Institute; SDM, Seed-based d Mapping; L, left; BA, Brodmann area*.

**Figure 2:**
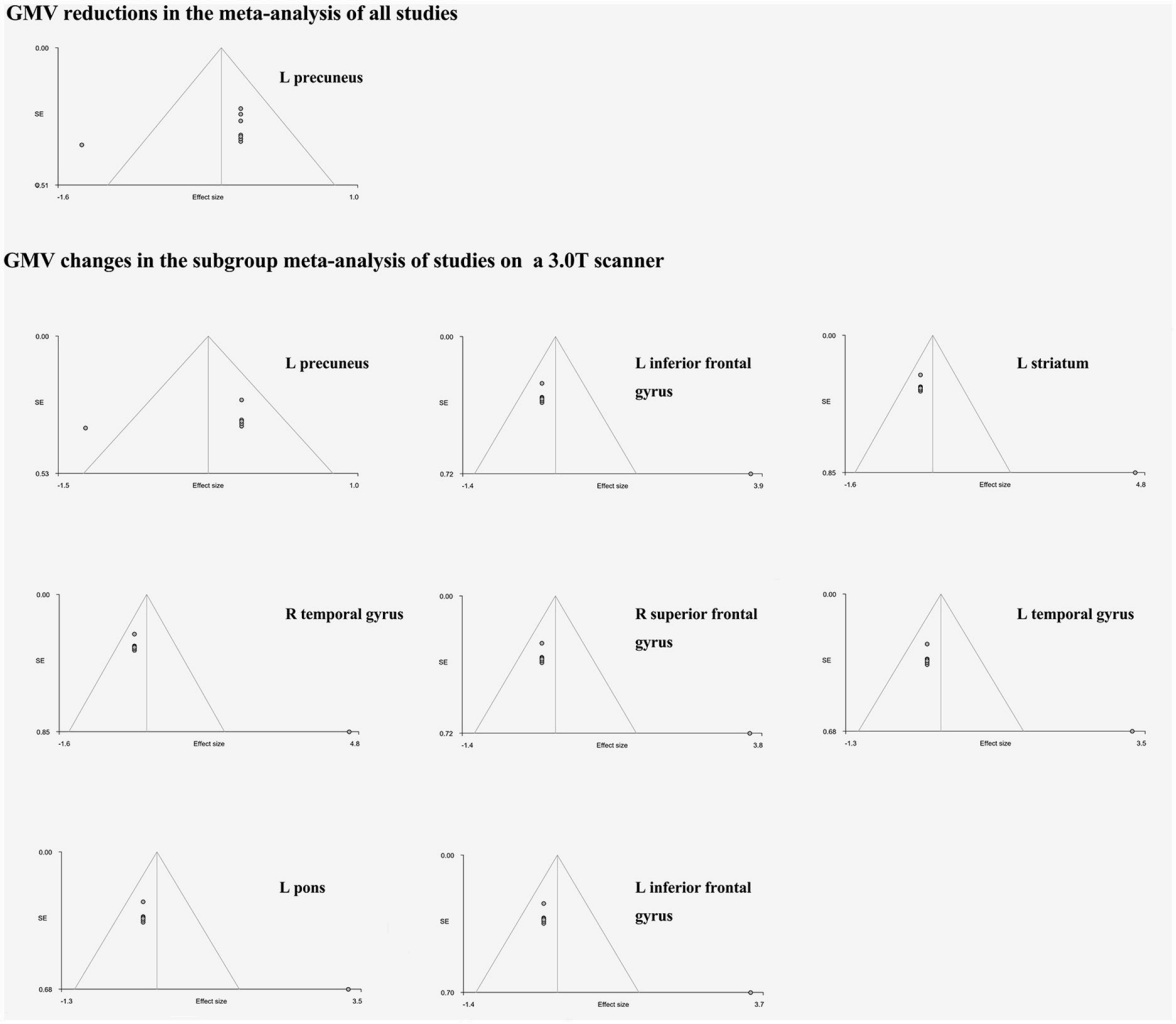
The funnel plots of the significant results of the whole meta-analysis and the subgroup meta-analysis. GMV, gray matter volume; L, left; R, right. The horizontal axis represents effect sizes of the significant peaks; The vertical axis represents the standard errors.

As two included studies with large patient samples were performed on a 1.5 T scanner and did not find significant results, there might be a potentially negative bias due to the less sensitivity of 1.5 T scanner to detect GM volume changes. Therefore, we conducted a subgroup meta-analysis including eight studies using a 3.0T scanner to avoid the negative bias. Besides the atrophy of GM in the left precuneus, the GM volume increases were found in a number of regions, including the bilateral frontal lobes, bilateral temporal lobes, left insula, left striatum and left pons (see details in Table 3 and Figure 2).

**Table 3:** The subgroup meta-analysis of studies performed on a 3.0T scanner: GM volume differences in ET patients relative to healthy controls.

**Regions**	**No. of voxels**	**Maximum MNI coordinates (x,y,z)**	**SDM-Z value**	***P*-value**	**Jackknife sensitivity**	**Egger test (*p*-value)**
**INCREASED GM VOLUME**						
L inferior frontal gyrus/ insula	294	−44,20,−8	1.030	0.0009	7 out of 8	0.005
L striatum/ olfactory cortex	228	−10,4,−16	1.057	0.0006	7 out of 8	0.003
R temporal lobe	106	46,−50,−4	1.056	0.0006	7 out of 8	0.003
R superior frontal gyrus	68	24,42,48	1.027	0.0009	7 out of 8	0.005
L temporal lobe	43	−40,−52,−8	1.008	0.0013	7 out of 8	0.006
L pons	41	−8,−18,−6	1.010	0.0012	7 out of 8	0.006
L inferior frontal gyrus	32	−38,18,22	1.020	0.0010	7 out of 8	0.005
**DECREASED GM VOLUME**						
L precuneus/L posterior cingulate gyrus, BA23	34	−10,−50,34	−1.538	0.0027	6 out of 8	0.053

*GM, gray matter; ET, essential tremor; No., number; MNI, Montreal Neurological Institute; SDM, Seed-based d Mapping; L, left; BA, Brodmann area*.

### Jackknife sensitivity analysis

The whole-brain Jackknife sensitivity analysis revealed that the significant GM volume reduction in the left precuneus (BA23) were replicable in eight out of 10 studies (see in Table 2). Subgroup meta-analysis on studies using 3.0 T scanner found significant GM volume increases in the abovementioned regions were replicable in seven out of 8 studies while the GM volume reduction in the left precuneus (BA23) remained significant in six out of eight studies (see in Table 3).

### Analyses of heterogeneity and publication bias

The heterogeneity analysis via the SDM software indicated a significant unexplained between-study variability of GM volume changes in the left cerebellar hemisphere, right caudate nucleus, right cingulate gyrus, left striatum, right cerebellar hemisphere and right inferior temporal gyrus (see details in Supplementary Table 1).

The funnel plots of all significant regional GM volume changes from both the whole meta-analysis and the subgroup meta-analysis were observed varied degrees of biases (see details in Figure 2). For statistical assessments, Egger's test was significant for the GM volume reduction in the left precuneus (*p* = 0.043), which survived in the subgroup meta-analysis (*p* = 0.053). However, the *P*-values of all GM volume increases in the abovementioned regions in the subgroup meta-analysis were far < 0.05 (Table 3).

### Meta-regression analyses

As illustrated in Table 4, the meta-regression analysis revealed that ET patients with higher mean age (available in all 10 studies) were positively associated with GM volume increases in the bilateral cerebellar hemispheres, left precentral gyrus, left temporal gyrus, right lingual gyrus, right occipital gyrus, right parietal gyrus, right frontal gyrus and with GM volume reductions in the bilateral precuneus, left cingulum, right supplementary motor area (SMA) and right insula. The ET groups with longer mean disease duration (available in all 10 studies) were positively associated with increased GM volumes in the bilateral cerebellar hemispheres. Additionally, ET patients with higher mean TRS scores (available in 6 studies) exhibited GM volume increases in the left cerebellar anterior lobules, bilateral SMA and GM volume decreases in the right cerebellar anterior lobule and left cerebellar posterior lobule. The percentage of patients with head tremor (available in five studies) was not associated with GM volume changes. However, these findings should be explained cautiously because they did not overlap clearly with significant results found in the whole meta-analysis or subgroup meta-analysis.

**Table 4:** Factors affecting GMV in ET patients compared with healthy controls.

		**Regions**	**No. of voxels**	**Maximum MNI coordinates**	**SDM-Z value**	***P*-value**
Effects of age (Elder patients compared with younger patients)	Increased GMV^*^:	R cerebellum, lobule VII, crus I/II	495	26,−76,−50	2.959	0.00032
		L precentral gyrus	98	−44,4,36	2.949	0.00033
		R lingual gyrus	89	30,−92,−14	2.923	0.00047
		R occipital gyrus	79	28,−84,34	2.923	0.00047
		L cerebellum, crus I	63	−24,−92,−26	2.923	0.00047
		R parietal gyrus	54	28,−54,46	2.923	0.00047
		R frontal gyrus	54	42,28,22	2.923	0.00047
		L temporal gyrus	51	−42,−66,10	2.927	0.00039
	Decreased GMV:	R insula	920	38,−2,10	−1.173	0.00137
		L precuneus/cingulum	422	−6,−50,36	−1.212	0.00077
		R SMA	233	10,6,50	−1.505	0.00001
		R precuneus	261	6,−46,64	−1.136	0.00206
Effects of disease duration (Patients with longer disease duration)	Increased GMV^*^:	R cerebellum	109	34,−54,−52	2.024	0.00327
		L cerebellum	51	−20,−88,−28	2.047	0.00296
Effects of disease severity (Patients have higher TRS scores)	Increased GMV:	L cerebellum, lobule III/IV/V	552	−4,−34,−26	2.047	0.00003
		L SMA	256	−10,−2,60	1.354	0.00157
		R SMA	208	10,2,54	1.408	0.00135
	Decreased GMV:	R cerebellum, lobule III/IV/V	462	20,−28,−24	−1.396	0.00040
		L cerebellum, lobule VIII	426	−26,−46,−56	−1.375	0.00061
Effects of head tremor (Studies with higher percentages of h-ET)		No significant results			

*GMV, gray matter volume; ET, essential tremor; No., number; MNI, Montreal Neurological Institute; SDM, Seed-based d Mapping, R, right; L, left; SMA, supplementary motor area; TRS, Fahn-Tolosa-Marin tremor rating scale; h-ET, ET patients with head tremor*.

* Only cluster with no. of voxels >50 are presented

## Discussion

To our knowledge, the current study is the first quantitative meta-analysis on VBM studies to identify the consistent whole-brain GM volume changes of ET patients relative to HCs. A small significant decreased GM cluster in the left precuneus extending to the left posterior cingulate gyrus (BA23) was found through meta-analysis.

Besides, a number of significant increased GM clusters located widely in the brain were found in the subgroup meta-analysis after excluding two studies which were performed on a 1.5T scanner with large samples and negative results. The results were largely replicable and stable after sensitivity and heterogeneity analyses. But unfortunately, the publication biases were detected in all these significant findings using either funnel plots or Egger's tests. Only GM volume reduction in the left precuneus extending to the left posterior cingulate gyrus (BA23) was the relatively unbiased. It should be noted that a excluded VBM study with absence of reported coordinates (13) did find significant GM volume reductions in the bilateral precuneus, which could have increased the robustness of the result in this region. Overall, GM volume reduction in the left precuneus extending to the left posterior cingulate gyrus (BA23) in ET patients was considered to be relatively reliable and consistent region by the current meta-analysis.

The precuneus, comprising a region of the default mode network (DMN) and the frontoparietal networks (35), was generally considered to play an essential role in the fundamental cognitive function (36). The importance of the precuneus has been demonstrated to have the highest resting metabolic rate within the DMN and widespread connectivity with the other regions of the DMN and frontoparietal networks (37, 38). The abnormality of the precuneus was detected widely in psychological disorders like depressive disorder (39) and was associated with some neurodegenerative diseases including Parkinson's disease (PD) (40, 41) and Alzheimer's disease (AD) (42). Moreover, the involvement of the precuneus in ET was also suggested by some other studies. Two studies using F-18-fluorodeoxyglucose positron emission tomography confirmed decreased glucose utilization in the precuneus in ET patients compared to HCs (43, 44). A functional MRI study based on a verbal working memory task detected increased connectivity between cerebellum and the precuneus in ET patients with low cognitive scores (45). Another resting-state functional MRI study also found the increased connectivity in the precuneus in ET patients relative to HCs and this alteration was positively associated with better performance on visuospatial ability and severer depressive symptoms (46). Another study (13) on ET patients also found a tended association between the smaller GM volume in the precuneus and the worse verbal fluency which is a common neuropsychological measure of executive function (47).

Another GM volume reduced cluster, the posterior cingulate cortex (PCC), was identified in the current study. PCC is a highly connected and metabolically active region as one of the core parts of the DMN (35) and plays a key role in the cognitive function such as memory and attention (48). A wide range of neurological and psychiatric disorders such as depressive disorder and AD also showed structural and functional abnormalities in the PCC (49, 50). However, there was rare report of the PCC abnormalities in ET patients. It was possibly because that only a small region of PCC was affected and the location was close to the precuneus. The current meta-analysis confirmed atrophy of GM in PCC in addition to the precuneus.

According to these above findings, the finding of the current study that GM volume reduction in the precuneus extending to the PCC might be linked to the cognitive dysfunction and depressive symptoms in ET patients, which are common non-motor manifestations of ET (51, 52). Although two (8, 10) of the ten included studies showed abnormalities in the precuneus and PCC in ET patients, cognitive disorders and depression were not reported in the ET patients, which may be due to the low sensitivity to mild cognitive impairments criteria (Diagnostic and Statistical Manual of Mental Disorders-IV criteria) used in the studies (8).

Recently, an increasing number of evidences from pathological, neuroimaging, physiological and clinical researches suggested the cerebellum was the neurological hallmark of ET (3–6). However, the current meta-analysis did not identify any region with GM volume abnormalities in cerebellum though quite a few included studies reported it after comparing generally ET patients or specific ET patients (e.g., with head tremor) with HCs. It indicated that these GM volume abnormalities found in different studies were heterogeneous and inconsistent, which was supported by the analysis of heterogeneity (Supplementary Table 1). The heterogeneity might result from the different patient groups with heterogeneous characteristics, as the meta-regression analyses indicated potential associations between GM volume abnormalities in different cerebellar subregions with the age, disease duration and the severity of tremor. The advancing age of ET patients was positively associated with increased volumes of the cerebellar lobule V and crus I/II. These subregions were considered to play roles in cognitive function and emotional control (53). It seemed like the cerebellar GM volume was of compensatory increase in the elder ET patients to resist the cognitive dysfunction and depression. However, what the role of normal aging was in this mechanism need further longitudinal investigations. The increased volume of cerebellar GM was associated positively with longer disease duration and higher TRS scores, which also indicated a potential compensatory mechanism. Some previous pathological and physiological studies (54, 55) reported the compensatory mechanism of cerebellum in ET. Further clinical studies can focus on it.

It should be pointed out that our finding of GM volume reductions in the precuneus and PCC were located only on the left side. A previous study reported the left lateralization of the GM volume decrease in the precuneus and PCC (10) but more studies found abnormalities of the precuneus or PCC in both sides (8, 13, 45) or in the right side (46). Such lateralization is difficult to explain. It may be due to the discrepancy of the characteristics of the patient samples because the asymmetry of tremor was associated with asymmetric pathological changes in ET patients though these pathological changes were mainly found in cerebellum (56). Another one in particular was that the meta-regression analyses revealed a potential link between GM volume reductions in the left precuneus/PCC and the age rather than the disease progression. There was lack of longitudinal study focused on the relationship between the precuneus/PCC and age. A previous pathological study (57) explored the biochemical alterations of the precuneus and PCC during normal aging. Most of bioindicators of emerging brain pathology remained steady in precuneus with advancing age. However, the bioindicators can not reflect the structural alterations and functional connectivity. Whether the structural lesion in this region is a specific pathophysiological change on ET or a natural aging process need further longitudinal investigations.

## Limitations

Several limitations should be acknowledged in the current study. First, studies from which we could not extract data and in languages other than English were not included, which might affect the significant results. Second, ET encompasses a range of heterogeneous clinical phenotypes, but we were not available to compare different phenotypes with HCs due to the lack of the original data. Besides, according to the new criterion, all included ET groups were diagnosed as “simple ET” instead of “complex ET” which showed additional neurological signs like impaired tandem gait, questionable dystonic posturing and rest tremor (2). Thus, our result only reflected a general alteration of GM volume in simple ET patients. There might be more abnormalities when the meta-analysis was focused on a single phenotype or included complex ET patients. Third, because of the incomplete information and heterogeneity of included studies, in the meta-regression analysis we did not examine the potential effects of some variables like the cognitive function and type of tremor, which might influence the findings of the current meta-analysis. Fourth, all of the included studies were cross-sectional studies that only reflected static alterations of GM volume in ET patients. Future longitudinal studies may provide continuous findings. Fifth, the alteration of the GM volume is a relatively later pathological manifestation in a neurodegenerative process and may not account for early impairments of the brain region. The future meta-analysis including functional MRI studies will be a great complimentary to our study. Finally, the heterogeneity of different methods in VBM studies, including pre-processing protocols, smoothing kernels, and statistical thresholding methods, cannot be ruled out entirely. The methodology of SDM that using reported coordinates rather than raw data also influences the accuracy (19).

## Conclusion

The current meta-analysis including VBM studies on ET patients suggested a relatively consistent and reliable GM volume reductions in the left precuneus and PCC, which might be linked to cognitive dysfunctions and psychological signs in ET. The consistent structural abnormalities were not detected in the cerebellum, but the meta-regression analyses revealed the effects of the age, disease duration and severity of tremor might disturb the findings in the cerebellum. Future studies might focus on more homogeneous ET patients (e.g., a single phenotype) and the role of the precuneus and PCC in ET pathophysiology also needs further investigations.

## Author contributions

QH: Literature search, data extraction, statistical analysis, manuscript writing; YH: Literature search, data extraction, statistical analysis review, and critique; HS: Research organization, manuscript review, and critique.

### Conflict of interest statement

The authors declare that the research was conducted in the absence of any commercial or financial relationships that could be construed as a potential conflict of interest.
