# Supplementary material for: A Voxel-Wise Meta-Analysis of Gray Matter Abnormalities in Essential Tremor
Source: Front Neurol. 2018 Jun 26;9:495. doi: 10.3389/fneur.2018.00495 (PMC6028592; doi:10.3389/fneur.2018.00495)
Supplement: Supplementary file 1 [file Table_1.DOCX]

| **Supplementary Table 1** Regions of GMV heterogeneity from the SDM analysis | | | | | |  |
| --- | --- | --- | --- | --- | --- | --- |
| Regions | No. of voxels | Maximum MNI coordinates  (x,y,z) | SDM-Z value | P value | | |
| Left cerebellar hemisphere | 326 | -24,-64,-42 | 3.761 | 0.000521 |  | |
| Right caudate nucleus | 238 | 12,12,20 | 3.580 | 0.000821 |  | |
| Right cingulate gyrus | 117 | 6,12,26 | 3.478 | 0.001048 |  | |
| Left striatum | 46 | -10,4,-16 | 3.195 | 0.002222 |  | |
| Right cerebellar hemisphere | 33 | 14,-66,-40 | 3.334 | 0.001533 |  | |
| Right inferior network | 16 | 46,-50,-4 | 3.178 | 0.002333 |  | |
| **Key:** GMV, gray matter volume; No., number; MNI, Montreal Neurological Institute; SDM, Seed-based d Mapping. | | | | | |  |
