# Supplementary material for: A Voxel-Wise Meta-Analysis of Gray Matter Abnormalities in Essential Tremor
Source: Front Neurol. 2018 Jun 26;9:495. doi: 10.3389/fneur.2018.00495 (PMC6028592; doi:10.3389/fneur.2018.00495)
Supplement: Supplementary file 2 [file Table_2.DOCX]

**Supplementary Table 2. Criteria for quality assessment of individual studies**

| **Category 1: Sample characteristics (10)** |
| --- |
| 1. Patients were evaluated with specific standardized diagnostic criteria (1) |
| 1. Important demographic data (age and gender) were reported with mean (or median) and standard deviations (or range)) (2) |
| 1. Healthy comparison subjects were evaluated to exclude psychiatric and medical illnesses and demographic data was reported (1) |
| 1. Important clinical variables (e.g. illness duration, TRS scores) were reported with mean (or median) and standard deviations (or range)) (2) |
| 1. Sample size per group ≧10 (1) |
| **Category 2: Methodology and reporting (10)** |
| 1. All neuroanatomic measurements were taken without considering group assignment or subject identity (1) |
| 1. Magnet strength at least 1.5T (1) |
| 1. MRI slice-thickness ≤ 3 mm and more than 1 slice was identified and traced (1) |
| 1. The acquisition and preprocessing techniques were clearly described so that they could be reproduced (1) |
| 1. Measurements were clearly described so that they could be reproduced (1) |
| 1. Coordinates were reported in a standard space unless there was no significant difference (1) |
| 1. Significant results are reported after correction for multiple testing using a standard statistical procedure (FDR, FWE or permutation-based methods) (1) |
| 1. Conclusions were consistent with the results obtained and the limitations were discussed (1) |
| **Total**  /15 |
